# Considerations for conducting systematic reviews: evaluating the performance of different methods for de-duplicating references

**DOI:** 10.1186/s13643-021-01583-y

**Published:** 2021-01-23

**Authors:** Sandra McKeown, Zuhaib M. Mir

**Affiliations:** 1 Bracken Health Sciences Library, Queen’s University, 18 Stuart Street, Kingston, Ontario K7L 2V5 Canada; 2 Departments of Surgery and Public Health Sciences, Queen’s University & Kingston Health Sciences Centre, 76 Stuart Street, Kingston, Ontario K7L 2V7 Canada

**Keywords:** Bibliographic databases, De-duplication, Duplicate references, Reference management software, Study design, Systematic review software, Systematic reviews, Synthesis methods

## Abstract

**Background:**

Systematic reviews involve searching multiple bibliographic databases to identify eligible studies. As this type of evidence synthesis is increasingly pursued, the use of various electronic platforms can help researchers improve the efficiency and quality of their research. We examined the accuracy and efficiency of commonly used electronic methods for flagging and removing duplicate references during this process.

**Methods:**

A heterogeneous sample of references was obtained by conducting a similar topical search in MEDLINE, Embase, Cochrane Central Register of Controlled Trials, and PsycINFO databases. References were de-duplicated via manual abstraction to create a benchmark set. The default settings were then used in Ovid multifile search, EndNote desktop, Mendeley, Zotero, Covidence, and Rayyan to de-duplicate the sample of references independently. Using the benchmark set as reference, the number of false-negative and false-positive duplicate references for each method was identified, and accuracy, sensitivity, and specificity were determined.

**Results:**

We found that the most accurate methods for identifying duplicate references were Ovid, Covidence, and Rayyan. Ovid and Covidence possessed the highest specificity for identifying duplicate references, while Rayyan demonstrated the highest sensitivity.

**Conclusion:**

This study reveals the strengths and weaknesses of commonly used de-duplication methods and provides strategies for improving their performance to avoid unintentionally removing eligible studies and introducing bias into systematic reviews. Along with availability, ease-of-use, functionality, and capability, these findings are important to consider when researchers are selecting database platforms and supporting software programs for conducting systematic reviews.

**Supplementary Information:**

The online version contains supplementary material available at 10.1186/s13643-021-01583-y.

## Background

As research in the form of systematic reviews and meta-analyses is increasingly pursued, evidence from methodological studies can help researchers carry out these, and other types of knowledge syntheses, more efficiently [1, 2]. Part of the process of conducting systematic reviews and other syntheses is to identify all studies meeting pre-determined eligibility criteria to answer a research question in order to assess the full scope of research evidence and reduce the risk of reporting bias [3]. Searching multiple bibliographic databases to identify all studies is imperative, as many databases contain unique references in addition to overlapping content [4–7]. Managing database overlap prior to screening the search results helps prevent researchers from screening the same references for inclusion or exclusion multiple times. This is no small feat as many comprehensive literature searches retrieve thousands of search results. Therefore, efficient and accurate methods for removing duplicate references are needed.

Various methods are available for identifying and removing duplicate references, otherwise referred to as “de-duplicating” or “de-duplication”. At the database platform level, search results retrieved from different databases available via Ovid, such as MEDLINE, Embase, and Cochrane Central Register of Controlled Trials (CENTRAL), can be de-duplicated using Ovid multifile search [8]. Reference management software such as EndNote, Mendeley, RefWorks, and Zotero have long provided de-duplication functionality, and researchers have previously reported utilizing such tools for this purpose [9–12]. More recently, software programs specifically developed to facilitate the production of systematic reviews and other syntheses are starting to offer de-duplication functionality, including both proprietary (e.g., Covidence and DistillerSR) and free options (e.g., Rayyan) [13, 14].

Currently, only anecdotal evidence exists about the performance of de-duplicating references in Covidence and Rayyan [15, 16]. Previous research has demonstrated that de-duplication in EndNote, Mendeley, and RefWorks was only partially successful; however, these programs may have improved their algorithms in recent years (EndNote has since been sold by Thomson Reuters to Clarivate Analytics) [17]. As such, a broad assessment of different methods for de-duplication is lacking. Therefore, the objective of this study was to evaluate the accuracy, sensitivity, and specificity of default de-duplication settings for Ovid multifile search and commonly used electronic methods, including EndNote desktop X9, Mendeley, Zotero, Covidence, and Rayyan.

## Methods

### Database searches

Database search strategies from a prior synthesis on the topic of psilocybin-assisted therapies were modified to collect a sample of references for this study. Psilocybin is a naturally occurring tryptophan derivative with psycho-active properties found in several species of mushroom [18]. While several psilocybin trials have demonstrated safety, tolerability, and efficacy in treating a range of mental health disorders, legal prohibition of psilocybin in many countries hinders continued clinical investigation and therapeutic use [18]. This search topic was within the scope of a variety of databases (biomedical and subject-specific) and allowed retrieval of a heterogeneous sample of references including a variety of publication types (articles, book content, and grey literature in the form of conference proceedings and dissertations), dating back to the 1950s, in over 10 languages.

The bibliographic databases selected to search for studies included the minimum three recommended for Cochrane Reviews (MEDLINE, Embase and CENTRAL), which are generally considered to be the most important sources to search for reports of trials [3, 4, 19–22]. Ovid is the only platform that offers access to all three of these databases (as opposed to other platforms such as EBSCO and ProQuest), which also allows users to utilize its de-duplication functionality when searching across different databases. The Ovid interface was used to search the three key databases, as well as the psychology and psychiatry database PsycINFO (available via Ovid), because of its relevance to the search topic.

Customized, database-specific searches were executed simultaneously in the following four databases via the Ovid platform in December 2018: MEDLINE, Embase, CENTRAL, and PsycINFO. The “use” command for each database segment code ensured that each database-specific search only retrieved results from the appropriate database (Table 1). The search approach was kept simple by searching for a single concept only (psilocybin), and yet fairly comprehensive for a systematic review search by using a combination of keywords and database-specific subject headings.

**Table 1:** Customized database search strategies utilizing keywords^a^ and database-specific subject headings on the Ovid platform

#	Searches	Results
1	psilocybin*.mp. or psilocybin/	3130
2	1 use ppez [MEDLINE segment]	895
3	psilocybin*.mp. or psilocybine/	3130
4	3 use emczd [Embase segment]	1672
5	psilocybin*.mp. or psilocybin/	3130
6	5 use psyh [PsycINFO segment]	449
7	psilocybin*.mp.	3130
8	7 use cctr [CENTRAL segment]	114
9	2 or 4 or 6 or 8	3130

^a^By default, Ovid searches for your keywords in the default multi-purpose (.mp) set of fields

*The use of an asterisk in the search strategy indicates *unlimited right-hand truncation*, which searches for variations on a word that are formed with different suffixes

/The forward slash denotes a subject heading search

### De-duplication

To evaluate the six different methods of de-duplication, a benchmark set of de-duplicated search results was created through manual review of each reference (manual abstraction). Detailed steps for performing the manual abstraction are provided in Table 2. Consistent with the duplicate detection research conducted by Rathbone et al., “[a] duplicate record was defined as being the same bibliographic record (irrespective of how the citation details were reported, e.g. variations in page numbers, author details, accents used or abridged titles)” [23] p. 3. If the same study reported their results in a conference abstract/paper as well as a journal article, these references were not considered duplicates because they have separate bibliographic records. Detailed steps for de-duplicating references using the default settings in Ovid multifile search, EndNote X9, Mendeley, Zotero, Covidence, and Rayyan are provided in Additional file 1. Some of these software programs have made information about their default de-duplicating algorithms openly available on their website [24–26]. For example, the EndNote X9 User Guide states that “[b]y default, references are considered duplicates if they have the same reference type (such as Journal Article or Book), and the Author, Year, and Title fields are identical” ([26] p. 1).

**Table 2:** Steps for performing the manual abstraction

1	The citation and abstract fields from the combined database search results on Ovid were exported in Excel Sheet format.
2	The Excel Sheet was sorted by publication title.
3	Any brackets preceding a publication title (used in Ovid to denote non-English content) were removed and the Excel Sheet was re-sorted by publication title.
4	Duplicates were identified manually and highlighted.
5	The Excel Sheet was then sorted by author.
6	Duplicates were identified manually and highlighted.
7	Abstracts were used in steps 4 and 6 above to verify duplicate references, as needed. In some cases, if abstracts were not available, the full-text articles were retrieved.
8	Unique references were moved into a separate Excel Sheet to serve as the benchmark set.

### Analysis and outcomes

The benchmark set was used as the reference when analyzing the de-duplication performance of Ovid multifile search and the different software programs. False negatives and false positives were identified and recorded for each method, where false negatives represent references incorrectly identified as non-duplicates and retained, and false positives represent references incorrectly identified as duplicate references and flagged for removal. The study outcomes of accuracy, sensitivity, and specificity were reported with 95% confidence intervals using the Clopper-Pearson exact method [27]. Accuracy was defined as the proportion of correctly identified references (duplicate and non-duplicate) in relation to the benchmark set, sensitivity referred to the proportion of correctly identified duplicate references, and specificity related to the proportion of correctly identified non-duplicate references. False positives from each de-duplication method were further described by publication type, year, and language. Proportions were compared using exact binomial probabilities to identify any significant differences between primary research vs. non-primary research publications. Statistical significance was reached at *p* < 0.05.

### Missing data

In some cases, it was necessary to retrieve the full-text publication of false-positive duplicate references to determine the publication type and language. When verification was needed, we were unable to obtain the full-text publications for 10 EndNote X9 (5%) and 2 Rayyan (4%) false positives.

## Results

**Fig. 1:**
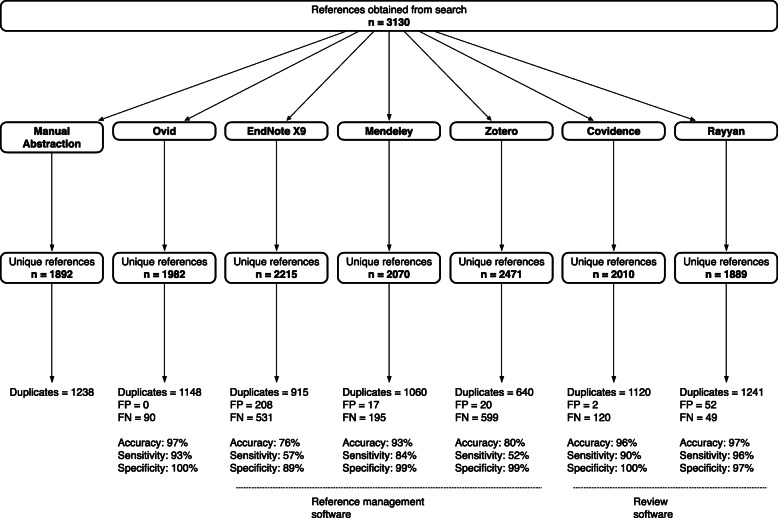
Unique references and duplicates removed using manual abstraction versus default algorithms for different de-duplication methods

The literature search strategy retrieved 3130 references in total from all four bibliographic databases on the Ovid platform (MEDLINE—895; Embase—1672; PsycINFO—449; CENTRAL—114). Following manual abstraction, the number of duplicates identified was 1238, leaving a benchmark set of 1892 unique, de-duplicated references (Fig. 1).

Following de-duplication using the default algorithm of each program, 1982 unique references remained in Ovid, after 1148 duplicates had been removed. Using reference management software, the numbers of unique references remaining after de-duplication were 2215 in EndNote X9 (915 duplicates), 2070 in Mendeley (1060 duplicates), and 2471 in Zotero (640 duplicates). Among review software, 2010 unique references remained in Covidence (1120 duplicates) and 1889 in Rayyan (1241 duplicates). Except Rayyan, all platforms identified fewer duplicates than the benchmark set obtained through manual abstraction (Fig. 1).

Compared to the benchmark set, all platforms failed to correctly identify certain duplicate references for exclusion (i.e., false negatives). The number of these false negatives ranged from 49 to 599, with the highest categorized in EndNote X9 and Zotero (Fig. 1). In comparison, the number of references incorrectly identified as duplicates for exclusion (i.e., false positives) showed a narrower spread, ranging from 0 in Ovid, to 208 in EndNote (Fig. 1). Using these results, we found that the most accurate methods for identifying duplicate references were Ovid, Covidence, and Rayyan (Table 3). Rayyan demonstrated the highest sensitivity for duplicate references, while Ovid and Covidence possessed the highest specificity (Table 3).

**Table 3:** Accuracy, sensitivity, and specificity of default algorithms for each de-duplication method, presented with 95% confidence intervals

	Accuracy	Sensitivity	Specificity
Ovid multifile search	0.97 (0.96, 0.98)	0.93 (0.91, 0.94)	1.00 (0.99, 1.00)
EndNote X9	0.76 (0.75, 0.78)	0.57 (0.54, 0.60)	0.89 (0.88, 0.90)
Mendeley	0.93 (0.92, 0.94)	0.84 (0.82, 0.86)	0.99 (0.986, 0.995)
Zotero	0.80 (0.79, 0.82)	0.52 (0.49, 0.54)	0.99 (0.98, 0.993)
Covidence	0.96 (0.95, 0.97)	0.90 (0.89, 0.92)	1.00 (0.996, 1.00)
Rayyan	0.97 (0.96, 0.974)	0.96 (0.95, 0.97)	0.97 (0.96, 0.98)

Lastly, we specifically analyzed the false-positive references marked for exclusion by each de-duplication method by publication date, language, and type. There was no clear trend noted in the analysis of these references by year of publication (data not shown). However, it became clear that most of these references were for English language publications, ranging from 85 to 100% (Table 4).

**Table 4:** False positives from default algorithms for each de-duplication method by language

	English	Non-English
Ovid multifile search	0	0
EndNote X9	190/208 (91%)	18/208 (9%)
Mendeley	16/17 (94%)	1/17 (6%)
Zotero	19/20 (95%)	1/20 (5%)
Covidence	2/2 (100%)	0/2 (0%)
Rayyan	44/52 (85%)	8/52 (15%)

**Fig. 2:**
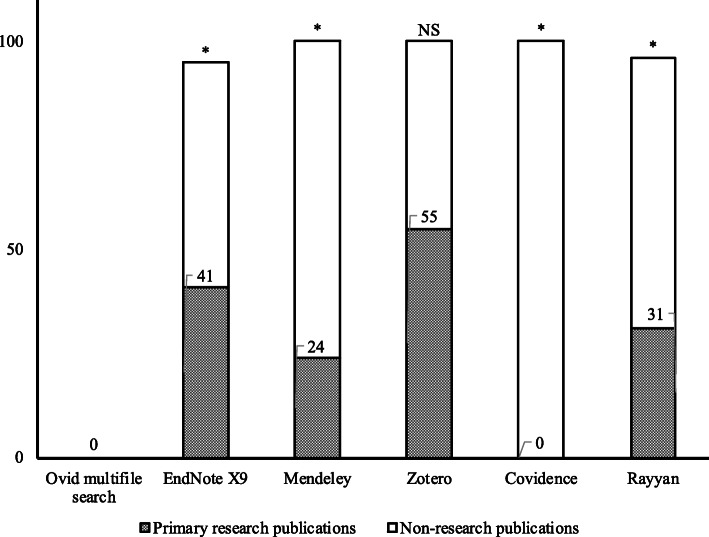
False positives from default algorithms for each de-duplication method by publication type. Numbers within the columns indicate the percentage of false-positive references from primary research publications. NS = not significant. * = significant (*p*-value < 0.02)

We then categorized the false positives by publication type, with the goal of identifying the number of excluded references that reported on primary research, and further classified these references as either full-text articles or conference abstracts/proceedings. When the bibliographic information provided by the database(s) could not be used to verify if primary research was reported, the full-text article itself was retrieved and reviewed wherever possible. Non-primary research publications included secondary studies, reviews, editorials, opinion pieces, etc. Except Covidence, false positives flagged by all the de-duplication methods included references that reported on primary research; these included 85/208 in EndNote X9, 4/17 in Mendeley, 11/20 in Zotero, and 16/52 in Rayyan (Table 5, Fig. 2). In Zotero, there was no significant difference between the proportions of false-positive primary research publications and non-research publications; however, in EndNote X9, Mendeley, and Rayyan, the proportion of false-positive non-research publications was significantly greater than primary research publications.

**Table 5:** False positives from default algorithms for each de-duplication method by publication type

	Primary research publications			Non-primary research publications
	Full-text articles	Conference proceedings/abstracts	All	
Ovid multifile search	0	0	0	0
EndNote X9^a^	81/208 (39%)	4/208 (2%)	85/208 (41%)	113/208 (54%)
Mendeley	3/17 (18%)	1/17 (6%)	4/17 (24%)	13/17 (76%)
Zotero	1/20 (5%)	10/20 (50%)	11/20 (55%)	9/20 (45%)
Covidence	0/2 (0%)	0/2 (0%)	0/2 (0%)	2/2 (100%)
Rayyan^b^	11/52 (21%)	5/52 (10%)	16/52 (31%)	34/52(65%)

^a^Unable to retrieve full-text publication for 10 false-positive duplicate references within EndNote X9

^b^Unable to retrieve full-text publication for 2 false-positive duplicate references within Rayyan

## Discussion

Researchers should consider utilizing electronic methods for de-duplicating search results to expedite the review process while being careful not to introduce a new source of bias through unintended exclusion of eligible studies. In this study, we found that the accuracy of default de-duplication algorithms in Ovid multifile search (97% [96–98]), Covidence (96% [95–97]), and Rayyan (97% [96–97.4]) significantly outperformed reference management software: EndNote desktop X9 (76% [75–78]), Mendeley (93% [92–94]), and Zotero (80% [79–82]). The specificity of different de-duplication methods was of particular interest though, since removing false positives from the screening process may result in missing eligible studies and introduce bias to syntheses. Among these, the exclusion of primary research studies may be particularly detrimental to evidence syntheses. Incomplete identification of relevant studies can also result in discrepancies between systematic reviews, which diminishes the usefulness of producing high-level research evidence in the first place: “navigating through these discrepancies can be demanding even for investigators who are well trained in evidence-based medicine and highly conversant on these methods” ([2] p. 492, [28]).

This study replicated previous research findings where using Ovid multifile search was the only de-duplication method that did not result in false positives, while also retaining a lower number of false negatives, comparatively [17]. Ovid may be able to circumvent false positives because the database platform has access to more bibliographic information for each record than what is exported to software programs (often just the citation and abstract information). Research teams with access to the three minimum databases recommended for Cochrane Reviews (MEDLINE, Embase, and CENTRAL) on the Ovid platform (as opposed to using PubMed or Wiley for example) can safely and effectively de-duplicate search results using Ovid multifile search [3]. However, this may still be of limited usefulness if additional bibliographic databases on other platforms such as EBSCO, ProQuest, or Web of Science will be searched to identify studies as well. In such cases, research teams may still benefit from pre-emptively de-duplicating search results retrieved from Ovid databases before exporting the search results to a reference manager or review software program. This may be particularly efficient for research teams who otherwise use reference management software for the de-duplication process, as these programs retained the highest number of false negatives compared to systematic review software. Proper utilization of Ovid multifile search is highly technical. Result sets which contain more than 6000 references cannot be de-duplicated; however, there are workarounds such as batching results into smaller sets using publication date ranges, for example. Working with a librarian can help researchers develop and execute complex and comprehensive database searches and is a best practice recommendation by international review collaborations [3, 29, 30].

After Ovid, Covidence derived the lowest number of false positives. The 2 false positives derived by this systematic review software program were publication types that did not contain original research (an editorial and book volume). It is worth noting that two of the three de-duplication methods with the highest specificity are subscription-based, proprietary options: Ovid (100% [99–100]) and Covidence (100% [99.6–100]). The default de-duplication settings in the other subscription-based, proprietary option (EndNote X9), was outperformed in specificity as well as accuracy and sensitivity by the three free-to-use options (Mendeley, Zotero and Rayyan).

Another considerable strength of de-duplicating references using Ovid multifile search or Covidence is that the process is fully automated, so duplicate references are automatically removed from the unique references and user mediation was not necessitated. In both cases, it is still possible to review which references were identified and removed as duplicates to look for false positives. However, this potentially time-consuming task is presumably not necessary in Ovid, since this de-duplication method has not been shown to derive false positives and may not be worthwhile in Covidence, which derived only 2 false positives that did not consist of original research in this study. Even so, if researchers decide to review duplicates in Covidence, they have the option to change its status to “not a duplicate,” which moves the reference over to the set of unique references to be screened.

Rather than employing full automation, the other four de-duplication methods evaluated in this study (EndNote X9, Mendeley, Zotero and Rayyan) have been designed to employ user oversight to guide decision-making for duplicates that are not exact matches. However, EndNote X9 allows users to merge duplicates as a batch rather than reviewing each potential duplicate individually. We do not recommend this approach as it derived the highest number of false positives in the present study. To maintain the highest possible recall in EndNote X9, researchers should consider utilizing a validated method for optimizing the de-duplication configuration, or confirming potential duplicates references individually [32]. The latter becomes problematic for researchers when they are left to confirm hundreds or thousands of potential duplicate references, in which case confirming each duplicate may be more work than just screening them all. It would be ideal if a newer version of EndNote X9 could improve the default de-duplication settings, since some researchers may not recognize the need to optimize the configuration, and this may result in unintentionally removing eligible studies. In regard to the critical appraisal of systematic reviews, it can be difficult for readers to detect if EndNote de-duplication methods may have introduced bias into systematic reviews because many researchers do not explicitly state whether the default settings or the optimized configuration was utilized.

To facilitate user oversight in the process of merging potential duplicates in Mendeley and Rayyan, both programs organize potential duplicates by varying confidence percentages. Future research could compare the efficiency of software programs that embed user oversight and decision-making into the de-duplication process and whether providing confidence percentages expedites the process.

In addition to the de-duplication methods studied here, researchers have made de-duplication modules freely available including Metta and SRA-DM (Systematic Review Assistant – Deduplication Module) [31, 32]. These modules have been criticized for being impractical because they require uploading large files to an online platform [31], which may partially explain why few systematic reviews report using these programs. Limited functionality in each of these modules prevented them from being evaluated in this study; Metta is designed for researchers who search MEDLINE via PubMed and not Ovid, and SRA-DM is designed for de-duplicating search results of less than 2000 references.

Strengths of the present study include evaluating the performance of the de-duplication process in systematic review software programs and the reference manager Zotero for the first time, as well as being the first study to analyze the characteristics of false positives derived from different de-duplication methods. Study limitations include using a sample of references that were retrieved from databases on the Ovid platform only. References exported from other search platforms (PubMed, EBSCO, ProQuest, Wiley, etc.) may behave differently. It was not possible to evaluate the new version of RefWorks in this comparison because the reference manager was unable to download one of the pre-saved sets of 1000 references exported from Ovid (the RIS file was too large at 5584 KB). This evaluation of default de-duplication algorithms does not consider user oversight processes built into some of the software programs. For this reason, the performance of DistillerSR was not compared in this study, as support staff for this proprietary systematic review software expressed that the necessity of user oversight built into their program would render an inequitable comparison to fully automated processes in programs like Covidence [33]. This research was conducted between December 2018 and January 2020 and the findings may become outdated as software programs are updated, or new versions become available. Research into whether de-duplication performance is impacted by different subject/topical searches is lacking and further investigation is needed in this area.

## Conclusions

This research demonstrates how well default algorithms for various de-duplication methods perform and provides strategies for improving their performance. These important considerations can help prevent researchers from unintentionally removing eligible studies and introducing bias into evidence syntheses. Two of the three de-duplication options with the highest specificity in this study (Ovid and Covidence) were also the most efficient methods, as they were fully automated. Electronic de-duplication methods that build in user oversight for merging duplicate references presumably perform better when users review and confirm which references are true duplicates, but this may be very time intensive. When choosing database platforms and software programs to facilitate the review process, researchers should consider de-duplication performance in combination with the availability and performance of other program functionalities such as screening references, resolving conflicts and extracting data.

## Supplementary Information

**Additional file 1.** Steps for performing the manual abstraction and de-duplicating references using default settings in various programs

## Data Availability

The datasets generated and analyzed during the current study are openly available in the Scholars Portal Dataverse repository, licensed under a Creative Commons Attribution-ShareAlike 4.0 International License, [10.5683/SP2/5TNOZ4].

## Abbreviations

CENTRAL: Cochrane central register of controlled trials

## References

[1] Page MJ, Shamseer L, Altman DG, Tetzlaff J, Sampson M, Tricco AC (2016). Epidemiology and reporting characteristics of systematic reviews of biomedical research: a cross-sectional study. Plos Med.

[2] Ioannidis JPA (2016). The mass production of redundant, misleading, and conflicted systematic reviews and meta-analyses. Milbank Q..

[3] Higgins JPT, Thomas J, Chandler J, Cumpston M, Li T, Page MJ, Welch VA. Cochrane Handbook for Systematic Reviews of Interventions version 6.1 (updated September 2020). Cochrane, 2020. Available from www.training.cochrane.org/handbook.

[4] Woods D, Trewheellar K (1998). Medline and Embase complement each other in literature searches. Br Med J.

[5] Topfer L-A, Parada A, Menon D, Noorani H, Perras C, Serra-Prat M (1999). Comparison of literature searches on quality and costs for health technology assessment using the MEDLINE and Embase databases. Int J Technol Assess Health Care.

[6] Biarez O, Sarrut B, Doreau CG, Etienne J (1991). Comparison and evaluation of nine bibliographic databases concerning adverse drug reactions. DICP Ann Pharmacother.

[7] Suarez-Almazor ME, Belseck E, Homik J, Dorgan M, Ramos-Remus C (2000). Identifying clinical trials in the medical literature with electronic databases: MEDLINE alone is not enough. Control Clin Trials.

[8] Advanced search techniques [Version 03.22.00]. Ovid Technologies, Inc. Web site. https://resourcecenter.ovid.com/site/help/documentation/ospa/en/Content/syntax.htm. Accessed 17 Mar 2020.

[9] Gomes F, Bergeron G, Bourassa MW, Dallmann D, Golan J, Hurley KM, et al. Interventions to increase adherence to micronutrient supplementation during pregnancy: a protocol for a systematic review. Ann N Y Acad Sci. 2020. 10.1111/nyas.14319. 10.1111/nyas.14319 PMC7384083 32052867

[10] Khan F, Rahman A, Carrier M, Kearon C, Schulman S, Couturaud F (2017). Long-term risk of recurrence after discontinuing anticoagulants for a first unprovoked venous thromboembolism: protocol for a systematic review and meta-analysis. BMJ Open..

[11] Aly M, O’Brien JW, Clark F, Kapur S, Stearns AT, Shaikh I (2019). Does intra-operative flexible endoscopy reduce anastomotic complications following left-sided colonic resections? A systematic review and meta-analysis. Color Dis.

[12] Ouweneel DM, Schotborgh JV, Limpens J, Sjauw KD, Engström AE, Lagrand WK (2016). Extracorporeal life support during cardiac arrest and cardiogenic shock: a systematic review and meta-analysis. Intensive Care Med..

[13] Covidence systematic review software. Veritas Health Innovation, Melbourne, Australia. https://www.covidence.org. Accessed 17 Mar 2020.

[14] Ouzzani M, Hammady H, Fedorowicz Z, Elmagarmid A (2016). Rayyan—a web and mobile app for systematic reviews. Syst Rev.

[15] Kellermeyer L, Harnke B, Knight S (2018). Covidence and Rayyan. J Med Libr Assoc.

[16] Olofsson H, Brolund A, Hellberg C, Silverstein R, Stenström K, Österberg M (2017). Can abstract screening workload be reduced using text mining? User experiences of the tool Rayyan. Res Synth Methods.

[17] Kwon Y, Lemieux M, McTavish J, Wathen N (2015). Identifying and removing duplicate records from systematic review searches. J Med Libr Assoc.

[18] Shore R, Ioudovski P, McKeown S, Durmont E, Goldie C. Mapping psilocybin-assisted therapies: a scoping review. medRxiv. 2019; doi.org/10.1101/2019.12.04.19013896.

[19] Halladay CW, Trikalinos TA, Schmid IT, Schmid CH, Dahabreh IJ (2015). Using data sources beyond PubMed has a modest impact on the results of systematic reviews of therapeutic interventions. J Clin Epidemiol.

[20] Sampson M, de Bruijn B, Urquhart C, Shojania K (2016). Complementary approaches to searching MEDLINE may be sufficient for updating systematic reviews. J Clin Epidemiol.

[21] Sampson M, Barrowman NJ, Moher D, Klassen TP, Pham B, Platt R (2003). Should meta-analysts search Embase in addition to Medline?. J Clin Epidemiol.

[22] Bai Y, Gao J, Zou D, Li Z (2007). Is MEDLINE alone enough for a meta-analysis?. Aliment Pharmacol Ther..

[23] Rathbone J, Carter M, Hoffmann T, Glasziou P. Better duplicate detection for systematic reviewers: evaluation of systematic review assistant-deduplication module. Syst Rev. 2015; 4(6). https://doi.org/10.1186/2046-4053-4-6. 10.1186/2046-4053-4-6 PMC4320616 25588387

[24] Covidence systematic review software. How does Covidence detect duplicates? Veritas Health Innovation, Melbourne, Australia. https://support.covidence.org/help/how-does-covidence-detect-duplicates. Updated 4 Feb 2019. Accessed 18 Mar 2020.

[25] Zotero. Duplicate detection. https://www.zotero.org/support/duplicate_detection. Updated 25 Nov 2017. Accessed 18 Mar 2020.

[26] Endnote Desktop X9. Find duplicate references. EndNote X9 Help User Guide. Philadelphia, Pennsylvania: Clarivate Analytics.

[27] Clopper CJ, Pearson ES (1934). The use of confidence or fiducial limits illustrated in the case of the binomial. Biometrika.

[28] Cook DJ, Reeve BK, Guyatt GH, Heyland D, Griffith L, Buckinghmam L (1996). Stress ulcer prophylaxis in critically III patients: resolving discordant meta-analyses. J Am Med Assoc.

[29] The Joanna Briggs Institute (2014). Joanna Briggs Institute reviewers’ manual: 2014 edition.

[30] Kugley S, Wade A, Thomas J, Mahood Q, Jørgensen AMK, Hammerstrøm K (2017). Searching for studies: a guide to information retrieval for Campbell systematic reviews.

[31] Bramer WM, Giustini D, de Jonge GB, Holland L, Bekhuis T (2016). De-duplication of database search results for systematic reviews in EndNote. J Med Libr Assoc.

[32] Jiang Y, Lin C, Meng W, Yu C, Cohen AM, Smalheiser NR. Rule-based deduplication of article records from bibliographic databases. Database (Oxford). 2014. 10.1093/database/bat086. 10.1093/database/bat086 PMC3893659 24434031

[33] Distiller SR. Evidence Partners Web site. 2020. https://www.evidencepartners.com. Accessed 17 Mar 2020.

