# Supplementary material for: Considerations for conducting systematic reviews: evaluating the performance of different methods for de-duplicating references
Source: Syst Rev. 2021 Jan 23;10:38. doi: 10.1186/s13643-021-01583-y (PMC7827976; doi:10.1186/s13643-021-01583-y)
Supplement: Supplementary file 1 — Additional file 1. Steps for performing the manual abstraction and de-duplicating references using default settings in various programs [file 13643_2021_1583_MOESM1_ESM.docx]

**Additional file 1**. Steps for de-duplicating references using default settings in various programs.

Ovid multifile search:

1. Customized database searches were executed in a simultaneous database search in MEDLINE, Embase, PsycINFO and CENTRAL on the Ovid platform.
2. Each customized database search was limited to the appropriate database segment (PPEZ, EMCZD, PSYH and CCTR, respectively).
3. The four database segment searches were combined using the ‘OR’ Boolean operator.
4. The “deduplicate” option was selected for the combined search results.
5. De-duplicated references were exported in Excel Sheet format.

EndNote desktop X9:

1. The combined database search results on Ovid were exported in EndNote format.
2. The references were opened in a new EndNote desktop library.
3. Default settings were used to “find duplicates.”
4. All duplicates identified were selected and deleted as a group.
5. The de-duplicated references were exported to Excel.

Mendeley:

1. The combined database search results on Ovid were exported in RIS format.
2. The RIS files were imported to Mendeley.
3. Mendeley automatically de-duplicated a small number of references.
4. After selecting “Check for Duplicates,” all remaining items identified as potential duplicates were merged individually.
5. The de-duplicated references were exported in RIS format to an intermediary (Covidence) and then exported in CSV format to Excel. (Since Covidence automatically identifies and moves duplicates into a separate area, all references identified as duplicates were first marked “Not a duplicate”).

Zotero:

1. The combined database search results on Ovid were exported in RIS format.
2. The RIS files were imported to Zotero.
3. Viewing “Duplicate Items,” all items identified as potential duplicates were merged individually.
4. The de-duplicated references were exported in CSV format to Excel.

Covidence:

1. The combined database search results on Ovid were exported in RIS format.
2. The references were imported to Covidence with de-duplication occurring automatically.
3. The de-duplicated references were exported in CSV format to Excel.

Rayyan:

1. The combined database search results on Ovid were exported in RIS format.
2. The RIS files were imported to Rayyan.
3. Viewing “Unresolved,” all entries detected as potential duplicates were merged individually.
4. The de-duplicated references were exported in CSV format to Excel.
